# Preparation and Characterization of Dopamine-Modified Carbon Fiber Paper Composites for Gas Diffusion Layers

**DOI:** 10.3390/polym15163428

**Published:** 2023-08-16

**Authors:** Jiahua Ma, Xiangyu Chen, Xiaoshuai Sun, Chuanshan Zhao

**Affiliations:** State Key Laboratory of Biobased Material and Green Papermaking, Qilu University of Technology (Shandong Academy of Sciences), Jinan 250353, China; 13290394238@163.com (J.M.); colacxy@163.com (X.C.); 17861407148@163.com (X.S.)

**Keywords:** carbon fiber modification, PEMFCs, dopamine

## Abstract

Carbon fibers (CFs) cannot be directly used for the preparation of CF paper because of their chemically inert nature. Herein, the surface of CFs was modified using the spontaneous oxidative self-polymerization of dopamine. By taking full advantage of the spontaneous oxidation and self-polymerization properties of PD to maintain the maximum strength of CFs, a polydopamine-modified CF paper (PDA-CFP) with excellent performance was prepared using PD-modified CFs (PDA-CFs). This increased the proportion of hydrophilic functional groups on the surface of carbon fibers, increased the O/C ratio on the CF surface by 6 times, and improved the bond strength between the modified CF and the adhesive by making full use of the interaction force between polydopamine and PVA fibers. In this way, the primary properties of the CF paper were improved. Overall, the results showed that the dispersion of CF was considerably improved with dopamine modification. In addition, the primary physical properties of PDA-CFP were better than those of virgin CF paper (CFP-0). PDA-CFP exhibited a maximum tensile strength of 2.04 kN·m^−1^, a minimum resistivity of 0.06055 Ω·cm^−1^, and a minimum porosity of 72.4%. The tightness was increased by up to 12.1%.

## 1. Introduction

In recent years, the issue of energy scarcity has gained significant attention, leading to an increasing demand for green energy derived from environmentally friendly resources. As a result, proton-exchange membrane fuel cells (PEMFCs) have emerged as a promising and prominent energy source due to their environmentally friendly, efficient, and safe characteristics [1]. In PEMFCs, hydrogen and oxygen act as the main reactants, producing water as well as usable electrical energy and unused thermal energy as the primary by-products [2,3]. This property makes PEMFCs highly desirable, as they result in minimal pollution and emissions in terms of raw materials and end products.

Typically, PEMFCs consist of two main components, i.e., the bipolar plate and the membrane electrode assembly (MEA) [4]. The gas diffusion layer (GDL), a crucial part of the MEA [5], functions as a support material within the fuel cell and plays a vital role in transferring generated water and heat during fuel cell operation. Additionally, the GDL is responsible for electron transport and carries the catalytic layer. To meet the demands of GDL fabrication, the raw material should possess high permeability, high porosity, low resistivity, and excellent mechanical strength. Presently, candidate materials satisfying these criteria include carbon cloths, nonwoven fabrics, carbon black paper, and carbon fiber paper (CFP). However, carbon cloths feature a prolonged production cycle and a complicated preparation process, resulting in higher application costs, and nonwoven fabrics exhibit poor durability, low working strength, and limited stability as well as low safety. Furthermore, limitations in the carbon black paper collection process and the preparation of raw materials lead to reduced porosity and inferior mechanical strength [6]. On the other hand, CFP, made from short carbon fibers (CFs), demonstrates excellent electrical conductivity, good mechanical strength, and outstanding mass and heat transfer properties while maintaining relatively lower raw material costs, making it a favorable choice as a raw material for fabricating the GDL.

Due to the unique production process of CFs, few reactive functional groups are present on the CF surface because of their excellent characteristics and production process [7]. Additionally, the typical “surface-core” structure of CFs results in their low surface energy [8]. The inertness of the CF surface affects their further development and utilization. However, the above-mentioned drawbacks can be overcome with the modification of CFs using methods including surface oxidation [9,10], rare-metal treatment [11,12], plasma treatment [13], chemical vapor deposition [14], electrodeposition [15], and surface grafting modification [16,17,18]. However, these techniques have many disadvantages, such as high costs, high hazards, deterioration of fiber strength after treatment, and generation of contaminated waste liquids [19], making it necessary to develop a new and greener CF treatment method.

Dopamine, a class of catecholamines, is an endogenous nitrogen-containing organic compound with very rich amino group contents. Additionally, PD exhibits a strong adhesion [20]. Owing to its distinct properties, the dopamine monomer is sensitive to oxygen and quickly undergoes a spontaneous oxidation reaction when exposed to oxygen, resulting in discoloration. The self-polymerization of dopamine can occur under alkaline conditions, and the rate of polymerization will be affected by the pH value [21,22].

**Figure 1:**
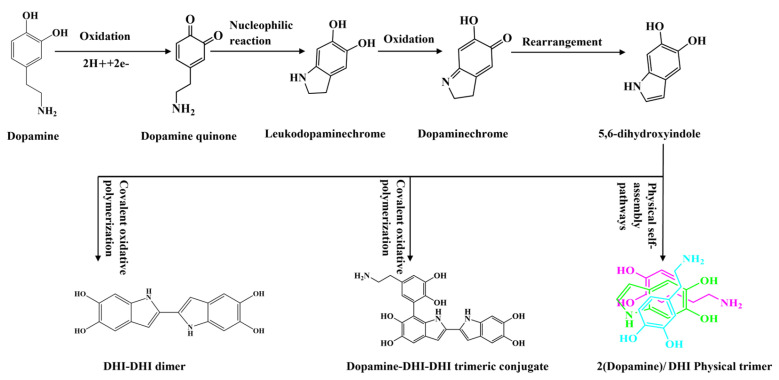
Dopamine oxidation mechanism process.

Dopamine is oxidized to produce polydopamine (PDA), a green biomolecule that can be used in various applications by virtue of its excellent water dispersibility and stability. Furthermore, PDA can undergo the self-polymerization reaction on CFs by forming abundant covalent and noncovalent bonds with the CF surface [23]. These characteristics are extremely effective for the preparation of CF paper, and the oxidation mechanism is shown in Figure 1.

Compared with the common dopamine functionalization of carbon fibers [24,25], this study uses a simpler and more convenient impregnation method for slurry preparation without the need for auxiliary processes such as stirring and ultrasonic agitation. This choice stems from the observation that stirring treatment during the practical fabrication of PDA-CFs demands precise control of rotation speed and stirring time. Inadequate settings in terms of these parameters could result in the agglomeration of carbon fibers into cohesive clusters, rendering subsequent dispersion challenging. Similarly, ultrasonic agitation, while effective in maintaining carbon fiber dispersion, imposes energy-intensive and equipment-demanding requirements due to the prolonged 24 h duration of dopamine treatment, requiring continuous water cooling. Additionally, this research incorporates a stepwise pre-treatment of carbon fibers involving both surface delamination and alkali treatment. Pre-treatment effectively eliminates impurities and organic residues from the carbon fiber surface, simultaneously activating it and providing additional active sites, facilitating dopamine interaction with the fiber surface. Moreover, pre-treatment allows fine-tuning of surface roughness and pore structure, thereby establishing a more suitable foundation for subsequent dopamine modification and promoting the adsorption and polymerization of dopamine molecules.

Based on a greener method of modifying the CF surface with dopamine, the good adhesion property of PDA and the abundant phenolic hydroxyl groups capable of forming hydrogen bonds with hydroxyl groups and carboxyl groups on the surface of pre-treated CFs was hereby used to prepare modified CFs. Additionally, the modified CFs were used for the preparation of CF paper. PDA was uniformly attached to the CF surface with dopamine modification and used as an intermediate to increase the CF–CF bonding ability and CF–polyvinyl alcohol (PVA) fiber bonding ability. The reactive groups on the surface of PD formed multiple hydrogen bonding and noncovalent bonding cross-linked networks with PVA fibers [26]. The hydrophilic functional groups greatly improved the dispersibility of CFs. Therefore, the CF paper prepared using modified CFs exhibited high strength and excellent uniformity. In addition, different sizing concentrations were hereby investigated, and the excellent performance of dopamine in preparing CF paper using CFs of different lengths was verified. The physical properties of CF paper were studied as well. Fabricating CF paper using polydopamine-modified CFs (PDA-CF) was found to exponentially increase the mechanical strength of CF paper while ensuring high porosity. Additionally, CF paper with excellent uniformity and high air permeability was produced without adding any dispersant.

## 2. Materials and Methods

### 2.1. Material

The materials used in this study include 3 mm and 6 mm polyacrylonitrile carbon fiber (Toray, Japan), PVA fiber (Shanghai Chenqi Chemical Co., Ltd., Shanghai, China), dopamine hydrochloride 98%, tris (hydroxymethyl) aminomethane 99.9% (Shanghai Maclean Biochemical Technology Co., Ltd., Shanghai, China), hydrochloric acid (Laiyang Economic and Technological Development Zone Fine Chemical Factory, Yantai, China), anhydrous ethanol (Tianjin Fuyu Fine Chemical Co., Ltd., Tianjin, China), and sodium hydroxide (Sinopharm Chemical Reagent Co., Shanghai, China).

### 2.2. Preparation of PD-Modified CFs

CFs were heated at a high temperature of 800 ℃ for 2 h, dried, and cooled. Then, the treated CFs were impregnated in a 15% sodium hydroxide solution for 0.5 h, washed, and vacuum dried to obtain pre-treated CFs (CF-1).

First, tris buffer was prepared [22]. Second, using the prepared tris buffer as the solvent, dopamine solutions with concentrations of 2 g/L, 5 g/L, and 8 g/L were prepared. Then, CFs were completely submerged in the dopamine solution and fully washed with deionized water after 24 h of sizing, followed by vacuum drying. The product was named PDA-X-CF, where X represents the concentration of dopamine solution used to treat the CF, namely, 2, 5, or 8.

### 2.3. Preparation of the Modified CF Paper

**Figure 2:**
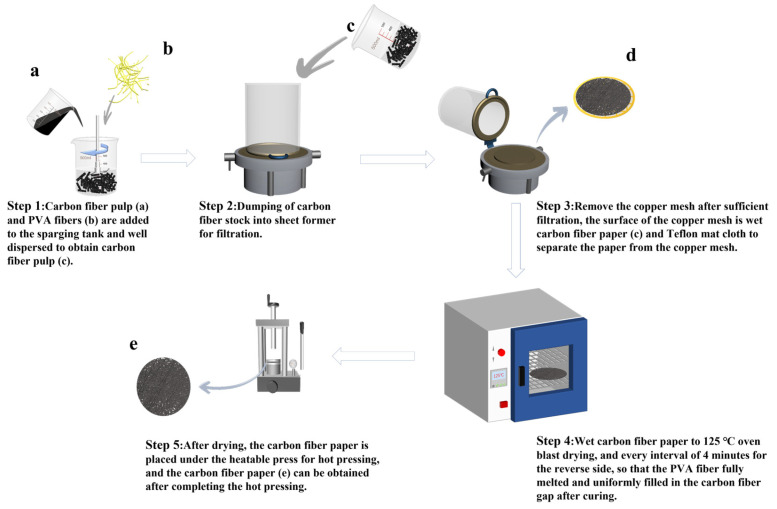
Carbon fiber paper preparation flow chart.

The preparation process of carbon fiber paper is shown in Figure 2. Modified CFs were dispersed in deionized water to prepare a CF pulp. Then, the CF pulp was mixed with an 8% PVA fiber. Stirring and dispersion of PVA-CF pulp were continued at room temperature to obtain a CF paper pulp. The CF pulp was added to the sheeting machine for full foaming and dispersion. Foaming was continued until there were no CF bundles, and immediate filtration and drainage were then performed. The CF paper was removed from the copper mesh using Teflon as the cloth liner. Subsequently, the wet paper web was placed in an oven for multiple reverse side de-watering and drying. The dried CF paper was subjected to a 24 h moisture balance operation at constant temperature and humidity. The CF paper was hot-pressed at 4 MPa and 170 °C for 4 min to obtain the final dopamine-modified CF paper (PDA-CFP). Finally, the physical properties of the CF paper and the optimal modification conditions were studied.

### 2.4. Characterization

Herein, the surface morphology and surface roughness of different CFs were characterized using scanning electron microscopy (SEM, Hitachi ultra-high-resolution field emission scanning electron microscope Regulus 8100, HITACHI, Japan) and atomic force microscopy (AFM, Multimode 8, Bruker Technology Co., Ltd., Beijing, China), and the surface functional groups of different CFs were characterized using Fourier transform infrared spectroscopy (FTIR). Meanwhile, the chemical compositions of different CF surfaces were investigated using X-ray photoelectron spectroscopy (XPS). Raman spectra of the different CFs used in this study were obtained using a Raman spectrometer, and X-ray diffraction (XRD) patterns were obtained using an X-ray diffractometer. The thermal stability of different CFs was explored using thermogravimetric analysis (TG), and mercuric pressure was used to obtain the thermal stability of various types of CFs. Furthermore, data on the porosity and pore size of carbon fiber paper were obtained using the mercuric pressure method. Finally, using a universal tensile machine, an air permeability tester, and a digital source meter, the physical properties of the carbon fiber paper such as tensile strength, stress–strain, permeability, and resistance were tested.

## 3. Results and Discussion

### 3.1. Surface Analysis of CF

**Figure 3:**
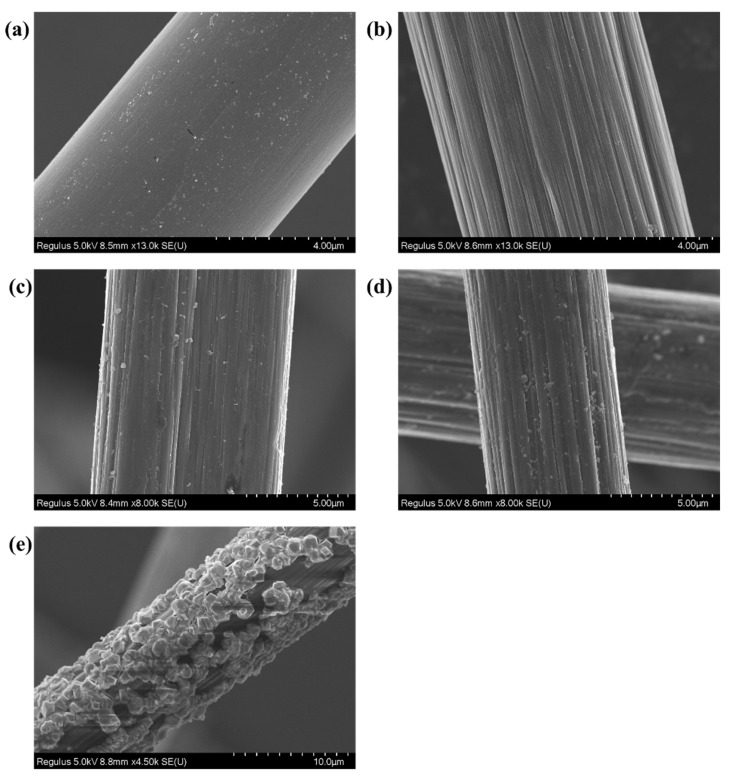
(**a**) SEM image of the virgin CF (CF-0) surface; (**b**) SEM image of the CF-1; (**c**) SEM image of the PDA-2-CF; (**d**) SEM image of the PDA-5-CF; and (**e**) SEM image of the PDA-8-CF.

As shown in Figure 3a, the surface of CF-0 was smooth, which was a protective agent wrapped on the surface of the carbon fiber by the manufacturer to protect the carbon fiber, and the glowing white spots were impurities on the CF-0 surface. As shown in Figure 3b, there were fewer impurities with obvious defective grooves and furrows on the surface of the CF-1, and this structure exposed more functional groups on the CF surface and facilitated subsequent dopamine sizing treatment. Figure 3c–e shows the occurrence of dopamine polymerization on the CF surface. As the dopamine concentration increased, the cluster size increased gradually. When CFs were treated with a dopamine sizing concentration of 2 g/L, PDA was uniformly dispersed in small clusters on the CF surface, and the longitudinal grooves of CFs were filled. When carbon fibers were treated with a dopamine sizing concentration of 8 g/L, the surface of CFs was covered with large PDA clusters of flocculation, and the surface grooves of the CF were almost completely filled. The images indicated that the sizing of CFs was complete after the dopamine self-polymerization reaction.

**Figure 4:**
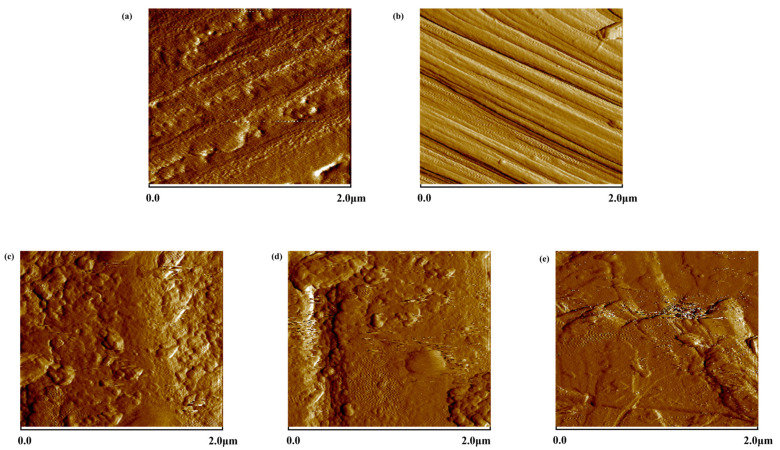
AFM topography of CF-0 (**a**), CF-1 (**b**), PDA-2-CF (**c**), PDA-5-CF (**d**), and PDA-8-CF (**e**) (2D).

**Figure 5:**
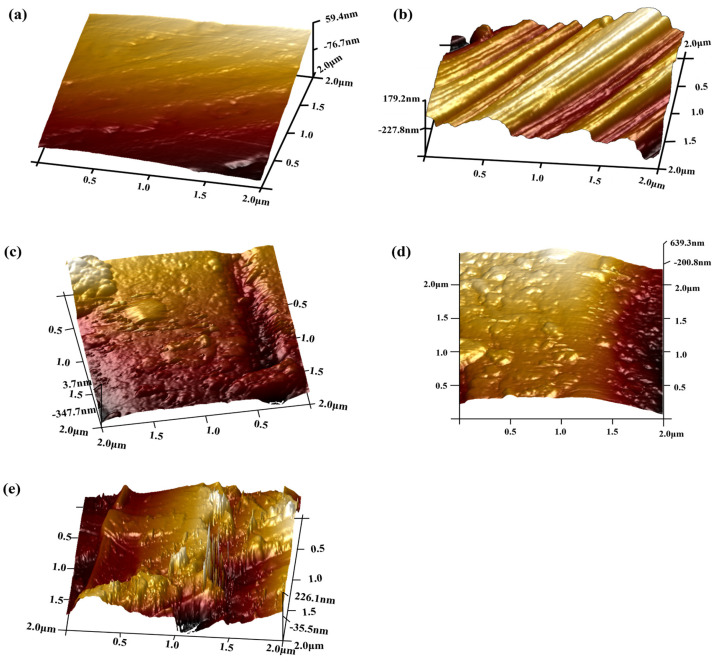
AFM topography of CF-0 (**a**), CF-1 (**b**), PDA-2-CF (**c**), PDA-5-CF (**d**), and PDA-8-CF (**e**) (3D).

From Table 1, it can be observed that after the pre-treatment to remove surface impurities, the Ra of the carbon fibers increased by nearly 1.8 times. This phenomenon is attributed to the removal of the resinous adhesive layer on the surface of carbon fibers with the pre-treatment, thereby exposing the original fiber surface. This process renders the carbon fiber surface rougher, as the removal of the resinous adhesive layer results in the emergence of more pronounced irregularities and minute cracks on the surface, thereby augmenting surface irregularity. With the augmentation of the dopamine coating concentration, both the Ra and Rq of the CFs significantly decreased. This reduction is attributed to the formation of a dense polydopamine coating on the carbon fiber surface as a result of dopamine treatment. This coating tends to fill micro-pores and fine crevices, rendering the surface smoother and more uniform, consequently reducing the Ra value. However, excessive concentrations lead to the clustering of dopamine into conglomerate structures on the carbon fiber surface. An analysis of Figure 4c–e and Figure 5c–e in conjunction with the Ra and Rq values of PDA-CF presented in Table 1 indicates that an increase in dopamine concentration results in a further increment in the surface roughness of carbon fibers. This phenomenon can be attributed to the formation of irregular polydopamine clusters on the carbon fiber surface. These findings jointly demonstrated the successful loading of dopamine onto carbon fibers, which resulted in notable changes in the surface morphology of the carbon fibers [27].

**Figure 6:**
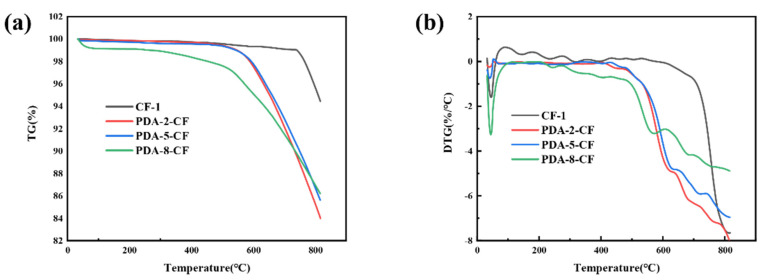
(**a**) TG curves for CF-1 and PDA-CF; and (**b**) DTG curves for CF-1 and PDA-CF.

As shown in Figure 6, a comprehensive analysis of the TG and DTG curves for the carbon fibers revealed that CF-1 experienced a noticeable weight change starting at 750 °C, while PDA-CF exhibited a significant weight change in the range of 580–610 °C. The presence of 8 g/L dopamine sizing concentration resulted in a higher accumulation of polydopamine on the carbon fiber surface, as observed in Figure 6, thus leading to a noticeable variation in the weight of PDA-8-CF with increasing temperature. The significant weight loss observed in PDA-CF indicated the successful attachment of dopamine onto the carbon fiber surface. Despite the fact that the thermal decomposition temperature of dopamine on the carbon fiber surface was higher than that of the original dopamine (150–250 °C) [28], it still decomposed at temperatures below 650 °C, suggesting that the polydopamine on the carbon fiber surface did not interfere with the subsequent carbonization and graphitization processes of the fibers.

### 3.2. Dispersibility of CFs

**Figure 7:**
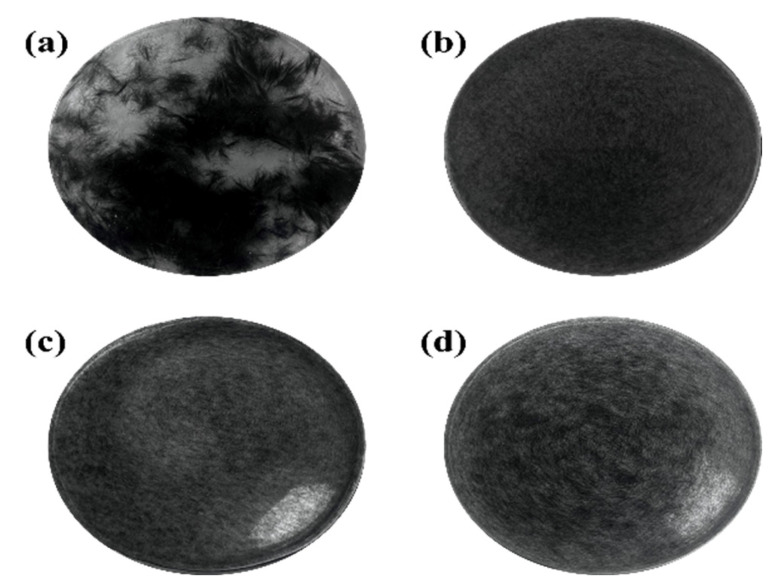
(**a**) Dispersions of CF-1; (**b**) dispersions of PDA-2-CF; (**c**) dispersions of PDA-5-CF; and (**d**) dispersions of PDA-8-CF.

Herein, CFs were prepared as 0.075% slurry, ultrasonically dispersed for 10 min, and placed in a white-bottom culture dish for 3 min to study the CF dispersion. The poor dispersion of the virgin CF resulted in poor stability after dispersion in water without dispersant (no significance as a control group). Therefore, CF-1 was chosen as a blank control sample. As shown in Figure 7a, CF-1 still dispersed poorly in water, in which there was pronounced flocculation of CFs. Figure 7b–d shows the dispersion of CFs after the three dopamine sizing treatments, respectively. Compared with CF-1, the CFs after dopamine modification exhibited excellent dispersibility in water, presenting almost no flocculation phenomenon, and could be uniformly dispersed in water. The abundant hydroxyl and amino groups in PD greatly improved the polarity of the surface of the CF and the hydrophilic property of the CF. Furthermore, the content of acidic functional groups on the CF surface increased after the modification. These functional groups considerably increased the negative charge on the CF surface after ionization in solutions, which in turn increased the repulsive force between the CFs. Under the multiple effects, the dispersibility of the CFs was greatly improved. The high sizing concentration caused the PD on the CF surface to react with the PD on the other CF surface. Additionally, the re-flocculation between fibers led to poor dispersion.

### 3.3. Performance Testing of CF Paper

Conventional CF-based paper requires the addition of papermaking agents such as dispersants and surfactants, and tedious steps such as the dilution of papermaking additives are required. However, modified CFs can overcome the disadvantages of CFs such as difficult dispersion. Meanwhile, PD has bonding properties, which can significantly reduce the amount of adhesive used [29]. Herein, the CF paper made from CF-0 was named CFP-0; the CF paper made from pre-treated CF was named CFP-1; and the CF paper made from dopamine-modified CF was named PDA-X-CFP (X represents different sizing concentrations).

#### 3.3.1. Homogeneity of the CF Paper

**Figure 8:**
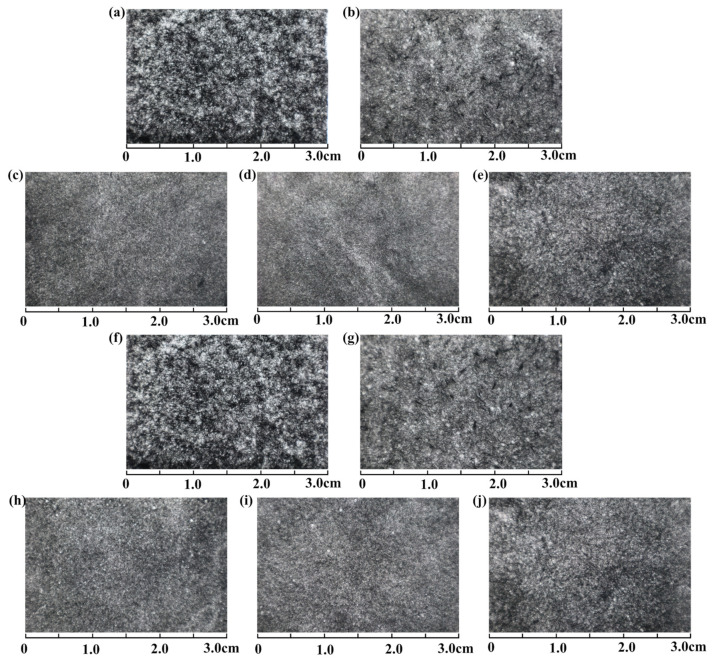
Light transmission renderings of CFP-0 (**a**), CFP-1 (**b**), PDA-2-CFP (**c**), PDA-5-CFP (**d**), and PDA-8-CFP (**e**) with a fiber length of 3 mm and light transmission renderings of CFP-0 (**f**), CFP-1 (**g**), PDA-2-CFP (**h**), PDA-5-CFP (**i**) and PDA-8-CFP (**j**) with a fiber length of 6 mm.

The uniformity of CF paper critically affects its mass transfer capacity [30]. The degree of flocculation and dispersion of CFs can be determined by comparing the CF paper uniformity. As shown in Figure 8, the dispersion degree of CFs directly affects the contact resistance of the fiber mass, while the uniformity of the CF dispersion can improve the strength of CF paper and facilitate the formation of uniform voids, exerting a considerable impact on the subsequent impregnation and curing of CF paper and carbonization [31,32]. CFP-0 exhibits a loose structure due to the inert nature of CF-0, resulting in uneven fiber dispersion and weak bonding between fibers. In contrast, pre-treatment removes impurities from the CF surface, enhancing dispersibility, reducing agglomeration of CF bundles, and improving light transmission and uniformity. Dopamine-treated CFs demonstrate excellent dispersibility, which leads to strong fiber bonding and uniformity after hot pressing. CF paper prepared with 3 mm CFs contains a percentage of large holes compared to CF paper prepared with 6 mm CFs, attributed to short fiber length and fewer cross-linking points. Under higher pressure during hot pressing, bonded fibers may fracture, affecting the macroscopic structure and light transmission of CF paper. However, the high intertwining of 6 mm fibers ensures minimal changes in the macroscopic structure even if breakage occurs between fibers under pressure.

#### 3.3.2. Tightness and Tensile Strength Performance of the CF Paper

**Figure 9:**
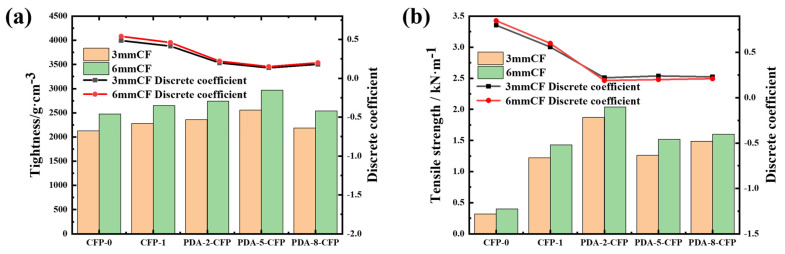
(**a**) Tightness of different CFPs and (**b**) tensile strength of different carbon fiber papers.

The thickness of CF paper directly affects its water transport behavior [29], with an excessive thickness increasing water saturation in the fuel cell and prolonging the time required to reach the water breakthrough point and the stable transport state of liquid water [33]. However, thin CF paper can address the issue, but it has a typically low mechanical strength, and enhancing the tightness between CFs can improve the paper’s strength and extend its service life. Tightness is the ratio of paper weight to thickness. The tightness of carbon fiber paper refers to the degree of closeness and the strength of bonding between the fibers. The tightness of carbon fiber paper reflects how densely the fibers are arranged and how well they are interconnected. For carbon fiber paper, an increase in tightness indicates stronger connections between fibers and a more compact fiber arrangement, resulting in excellent conductivity. Higher tightness generally positively affects the strength, abrasion resistance, and tensile performance of the paper. Figure 9a demonstrates that dopamine-modified CF paper (6 mm) exhibits a minimum tightness 2.67% higher than CFP-0, reaching 2969.697 g/cm^3^ compared with CFP-0’s 2475.248 g/cm^3^, which is a 12.12% increase. Furthermore, dopamine modifications introduce cross-linked structures and molecular bridges at fiber contact points. Dopamine molecules undergo self-polymerization to form dopamine polymer layers through condensation reactions. These layers create cross-linked bridges between fibers, increasing the number and strength of connections and resulting in a higher degree of tight fiber stacking.

The strength of CF paper is an important parameter when the paper is used as a GDL in practical applications [34]. Practically, CF paper is subjected to different degrees of force during transportation, loading, and use after preparation. Therefore, the mechanical strength of the CF paper is a guarantee of its longevity and performance as a substrate layer in GDLs in PEMFCs [35]. The tensile strength of the CF paper after various treatments is depicted in Figure 9b. Notably, irrespective of the CF length, the pre-treated CF paper exhibited superior tensile strength compared to CFP-0. Specifically, for 3 mm CF, the tensile strengths at different dopamine concentrations (low to high) were 1.87 kN·m^−1^, 1.26 kN·m^−1^, and 1.49 kN·m^−1^, respectively, which were 5.1, 3.8, and 4 times higher than the 0.32 kN·m^−1^ of CFP-0. Similarly, for 6 mm CF, the tensile strengths at different dopamine concentrations (low to high) were 2.04 kN·m^−1^, 1.52 kN·m^−1^, and 1.6 kN·m^−1^, respectively, which were 4.1, 2.8, and 3 times higher than CFP-0’s 0.4 kN·m^−1^.

The poor dispersion performance of CF-0 could be attributed to the presence of negative charges on the CF surface, which led to repulsive forces between the fibers and resulted in their clustering during dispersion. Moreover, the initial dispersion of CFs was challenging to maintain after re-flocculation, leading to poor paper uniformity and weak bonding between CF clusters.

In contrast, the pre-treatment effectively removed most of the impurities from the CF surface and increased the bonding force between CFs by enhancing the connections between exposed functional groups. The subsequent sizing treatment involved dopamine forming hydrogen bonds with PVA fibers, and the phenolic and amine functional groups in dopamine molecules underwent chemical reactions with the surface functional groups of carbon fibers, forming chemical bonds or physical adsorption. This chemical cross-linking enhanced the connections between fibers, consequently elevating the overall strength of the CF paper. Furthermore, the surface of the dopamine-modified carbon fiber exhibited certain adhesive properties and could interact with other fibers through physical adsorption, further promoting mutual adhesion and enhancing the overall strength of the paper, ultimately leading to a significant increase in the tensile strength of PDA-CFP.

#### 3.3.3. Porosity and Bulk Density of the CF Paper

**Figure 10:**
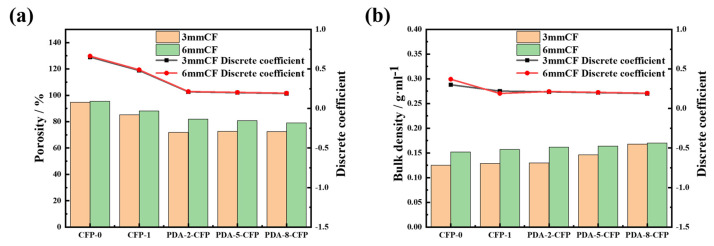
(**a**). Porosity of different CFPs and (**b**) bulk density of different CFPs.

Porosity can significantly determine the performance of the GDL [36]. A higher porosity leads to stronger drainage in the GDL substrate layer, a longer service life, a higher fuel cell energy conversion efficiency, and a better fuel cell performance [37]. Herein, the untreated CFs had poor dispersion uniformity, with a variation coefficient of 0.65. The porosity of the CF paper at the fiber aggregation was low, and the pore size was small (Figure 10a). By contrast, the low tightness of fiber binding and larger pores on the sparse CF resulted in high porosity, and the random distribution of the five measurement locations led to the large variation coefficient for porosity. The variation coefficient of the modified CF paper was 0.2, while that of the modified CF paper decreased by three-fold. The porosity of CFP-0 with different fiber lengths ranged from 94% to 95%, indicating poor dispersion of the original carbon fiber. After pre-treatment, the dispersion improved, leading to a decrease in porosity from over 90% to 80%. Dopamine treatment further enhanced dispersion, resulting in a more noticeable decrease in porosity. The carbon fiber paper prepared with 6 mm carbon fiber showed a less pronounced effect on porosity, maintaining it at approximately 80%, which indicated the greater impact of dopamine treatment on 3 mm carbon fiber. High porosity in carbon fiber paper was preferable for subsequent processing with conductive carbon materials. The dopamine-modified layer formed a polymer cover layer on the surface of the carbon fiber, which would fill the microscopic pores between the carbon fibers. The dopamine polymer in solution could form a colloidal substance, thereby forming a dense cover layer with the drying process and thus reducing the overall porosity of the carbon fiber paper. However, reducing the porosity might bring other advantages, such as improved paper compactness, mechanical strength, electrical conductivity, etc.

The volume density of carbon fiber paper mattered considerably, as it reflected its compactness and the presence of smaller pores and channels, which were beneficial for gas molecule collision and transfer and could enhance its electronic conductivity [34]. As shown in Figure 10b, the volume density of 6 mm CF paper was higher than that of 3 mm CF paper, which might be attributed to better fiber alignment, stronger fiber cross-linking, and reduced inter-fiber gaps in carbon fiber paper made from longer fibers. Furthermore, increasing the carbon fiber sizing concentration led to a gradual increase in the volume density of the carbon fiber paper. The volume densities of CFP-0 prepared from 3 mm and 6 mm carbon fibers were 0.125 g·mL^−1^ and 0.152 g·mL^−1^, respectively. After dopamine treatment, due to the chemical modification of the carbon fiber paper with dopamine, the volume density reached a maximum of 1.68 g·mL^−1^ for 3 mm CF paper and 1.7 g·mL^−1^ for 6 mm CF paper. The formation of a dense polydopamine coating on the carbon fiber surface filled micro-pores and fine gaps, resulting in a more compact overall structure and higher volume density for the carbon fiber paper.

#### 3.3.4. Contingency and Stresses of the CF Paper

**Figure 11:**
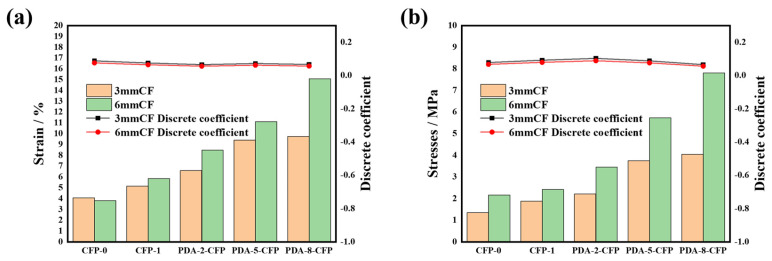
(**a**) Contingency of different CFPs and (**b**) stresses of different CFPs.

The stress-strain relationship of carbon fiber paper reveals the mechanical behavior and performance during the loading process. The stress-strain curve describes how the strain of the carbon fiber paper changes with stress under applied external loads. In this experiment, the endpoint of the stress-strain curve represents the paper’s fracture point, indicating that the paper can no longer withstand additional stress and will break. For clearer analysis, this study categorizes and compares both parameters. The strain in this study refers to the longitudinal strain of the carbon fiber paper, which is calculated by dividing the deformation length (ΔL) by the initial length (L0) when the paper is subjected to tension. The stress in this study refers to the normal stress of the carbon fiber paper, which is the external force acting on the unit area perpendicular to the paper’s cross-section. Its value is calculated by dividing the external force (F) by the cross-sectional area (A). As shown in Figure 11a, both the strain and stress of the CF paper showed significant changes compared with CFP-0, with a more pronounced effect observed for dopamine-treated longer carbon fibers. Regarding strain, the carbon fiber paper prepared with 3 mm carbon fibers presented a maximum strain of 9.73% after treatment, which was 2.3 times higher than the 4.06% of CFP-0. For the carbon fiber paper prepared with 6 mm carbon fibers, the strain of CFP-0 was 3.81%, which showed a 2.2-times increase to a minimum of 8.48% after dopamine treatment. The maximum strain reached 15.07%, showing a 3.9-times increase compared with CFP-0 [38]. Similarly, as depicted in Figure 11b, the stress of PDA-CFP was significantly higher than CFP-0. CFP-0 showed stresses of 1.34 MPa and 2.16 MPa, while the maximum stress increased to 4.04 MPa and 7.80 MPa after treatment, which was a nearly four-fold increase. The removal of impurities from the fibers during pulping facilitated compaction during paper preparation, resulting in a denser paper structure, which, to some extent, could enhance the stress–strain behavior of the carbon fibers. Moreover, the self-assembled polymer layer of dopamine on the carbon fiber surface enhanced the adhesion of the fibers with other components in the paper, allowing the fibers to better withstand stress. Additionally, dopamine modification might alter the chemical properties of the carbon fiber surface, such as increasing surface oxygen content and functional groups, thereby strengthening the interaction between fibers and other components, which contributed to the improvement in paper strength and stiffness. Consequently, dopamine modification greatly enhanced the stress–strain performance of the carbon fiber paper. 

#### 3.3.5. Resistivity and Permeability of the CF Paper

**Figure 12:**
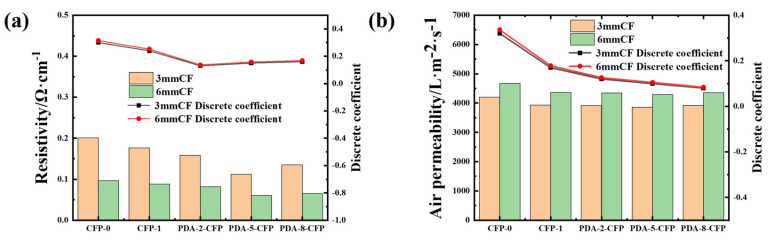
(**a**) Resistivity of different CFPs and (**b**) air permeability of different CFPs.

As shown in Figure 12a, the high resistivity of CFP-0 and the uneven distribution of resistivity at different sites (high resistivity coefficient of variation) were primarily attributed to uneven fiber dispersion, more fiber contact points at the flocculation, low resistivity in most of the contact area, less fiber in other void parts, loose structure, and high resistivity due to few electrons transport channels [29,39]. The introduction of dopamine modification led to a reduction in the resistivity and resistivity coefficient of variation for CFP-1 compared with CFP-0. The CFs exhibited a uniform dispersion after dopamine treatment, resulting in minimal variation in resistivity at different sites and a small resistivity variation coefficient. The resistivity reduction trend in the CF paper prepared with both lengths was similar to that of CFP-0 but with significantly improved performance. For 3 mm length fibers, with increasing dopamine concentration, the resistivity of PDA-CFP-2, PDA-CFP-5, and PDA-CFP-6 was measured as 0.1584 Ω·cm^−1^, 0.11215 Ω·cm^−1^, and 0.13536 Ω·cm^−1^, respectively, compared with CFP-1’s 0.17615 Ω·cm^−1^, indicating a reduction in resistivity by 10.07%, 36.33%, and 21.16%, respectively. Meanwhile, for 6 mm length fibers, with increasing dopamine concentration, the resistivity of PDA-CFP-2, PDA-CFP-5, and PDA-CFP-6 was measured as 0.8157 Ω·cm^−1^, 0.6055 Ω·cm^−1^, and 0.6457 Ω·cm^−1^, respectively, compared with CFP-1’s 0.8795 Ω·cm^−1^, suggesting a resistivity reduction of 7.25%, 31.15%, and 26.58%, respectively. Clearly, as the dopamine concentration increased, the resistivity initially decreased and then increased, with the optimal resistivity observed at the 5 g/L dopamine concentration for both 3 mm and 6 mm length fibers. The decrease in resistivity could be attributed to the increased functional group content on the surface of the carbon fiber after dopamine modification. The functional groups in the dopamine molecule chemically reacted with the carbon fiber surface, forming chemical bonds with a higher oxygen content and a polymer layer with a conjugated structure. This covering layer enhanced the electrical conductivity of the carbon fibers by providing more active sites for charge transfer and electron transport. Additionally, the increased roughness and surface activity of the carbon fiber surface induced with dopamine further contributed to the efficiency of electron transport and resulted in improved electrical conductivity of the dopamine-modified CF paper compared with CFP-0. While higher concentrations of dopamine might lead to overgrowth or aggregation of polymer layers, potentially affecting conductivity, the overall enhancement in electrical conductivity achieved with dopamine modification outweighed any negative effects, proving dopamine modification as an effective strategy for improving the electrical conductivity of CF paper.

As the base layer of the GDL, CF paper should possess high air permeability to enhance the transfer efficiency of different systems and improve fuel cell performance [40]. It can be observed in Figure 12b that the air permeability of the CF paper prepared using dopamine-modified carbon fiber showed a significant change. The air permeability of CFP-0 prepared with 3 mm and 6 mm length carbon fiber was 4230 L·m^−2^·s^−1^ and 4670 L·m^−2^·s^−1^, respectively. However, with an increase in dopamine concentration, the air permeability initially decreased and then increased. At a sizing concentration of 5 g/L, the air permeability for the 3 mm and 6 mm CF paper was measured to be 3861 L·m^−2^·s^−1^ and 4290 L·m^−2^·s^−1^, respectively, which was 8.72% and 8.13% lower than that of CFP-0, respectively. Subsequently, the air permeability increased again, which could be attributed to the following reasons: at lower dopamine concentrations, the polymerization reaction of dopamine molecules on the surface of carbon fibers was relatively limited, resulting in a thin modified layer and decreased porosity. However, as the concentration increased, the cohesive effect of dopamine polymers saturated, causing polymer particles to aggregate and form larger gel structures, which, in turn, created larger pore spaces on the fiber surface and led to an increase in porosity.

### 3.4. Mechanistic Analysis of Dopamine-Modified Carbon Fiber

**Figure 13:**
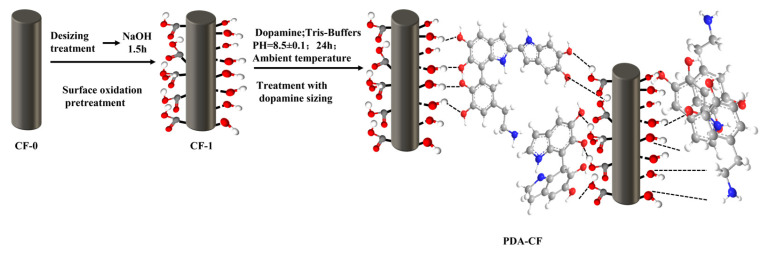
Schematic diagram of dopamine modified carbon fiber process.

Figure 13 shows the schematic diagram of the process of dopamine modification of carbon fiber. As can be seen from Figure 13, the carbon fiber surface modification process using dopamine involved impregnating the carbon fiber with dopamine and allowing the self-polymerization of dopamine on the carbon fiber surface to form polydopamine. This polydopamine contained abundant amino and hydroxyl functional groups, and numerous hydrogen bonds with the functional groups were formed on the carbon fiber surface. As a result, the surface of the carbon fiber became more hydrophilic, and the number of active groups increased, greatly enhancing its dispersibility. The combined effects of the chemical reactions and self-polymerization of dopamine resulted in a solid polymer coating on the carbon fiber surface, which further created a strong cross-linking structure and covalent bonding between the carbon fibers, leading to improved bonding strength among the fibers. This modification significantly enhanced the mechanical strength and stability of the carbon fiber paper while also improving its electrical conductivity and surface activity. Overall, the use of dopamine as a surface modifier effectively improved the performance of carbon fiber paper in various applications.

**Figure 14:**
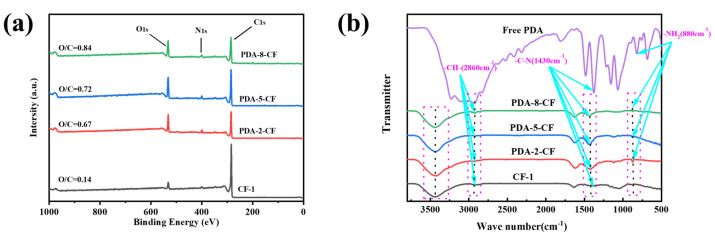
(**a**) The ratio of O/C contents in CFs after different treatments and (**b**) the combined FTIR spectrum of different treated CFs.

Figure 14a shows that the CF surface primarily comprised carbon (284.82 eV) and oxygen (532.48 eV), and the nitrogen (399.8 eV) content was rather insignificant [41]. The appearance of the nitrogen peak was attributed to the fact that some impurities were not completely removed during the pre-treatment of the CFs. After dopamine polymerization, the elemental ratio on the CF surface changed considerably, accompanied by the increased nitrogen (399.8 eV) content. The O/C ratio on the CF surface became larger with increasing dopamine concentration. The O/C ratio was proportional to the amount of oxygen-containing functional groups present on the CF surface [42]. The change in the O/C ratio of the CF surface with the dopamine concentration confirmed the successful polymerization of dopamine on the PDA-CF surface.

Figure 14b shows a comparison between the FTIR spectra of pre-treated CFs and sized CFs. Simultaneously, the FTIR spectrum of free dopamine was used as a comparative reference, aligning with the characteristic peaks of free dopamine to further substantiate the occurrence of dopamine oxidative self-polymerization on the carbon fiber surface. After an analysis of distinctive peaks in the spectrum of free dopamine, evident characteristic peaks were observed at -NH_2_ (880 cm^−1^) and C-N (1430 cm^−1^). Considering the -OH stretching vibration on the CF surface, the FTIR spectra of all CFs exhibited peaks near 3450 cm^−1^. The peaks in the FTIR spectra of the dopamine-modified CFs underwent redshifts in varying degrees because of the presence of -NH_2_ on the surface of PDA-CF (-NH_2_ has stretching vibration at 3300–3500 cm^−1^) [43]. The FTIR spectra of all CFs showed peaks at 1655 cm^−1^, 2850 cm^−1^, and 2930 cm^−1^, corresponding to the presence of C=C, -CH_2_, and -CH_3_ stretching vibrations in the CF structure [44], respectively. Additionally, the intensity of the -CH- stretching vibration peak at 2860 cm^−1^ increased considerably, which could be explained by the hydrogen bonding between dopamine and CF molecules and the dopamine-induced C=C to C-C transition of some CFs during the oxidative self-polymerization reaction. Furthermore, the FTIR spectra of CFs after sizing treatment presented distinct vibrational peaks at 1400 cm^−1^ and 430 cm^−1^ because of the superposition of the bending vibration in the -OH plane of PDA-CF (1410 cm^−1^) and the C-N (1430 cm^−1^) stretching vibration. Regarding the change in CF after sizing at 880 cm^−1^ [45], a sharp peak at 880 cm^−1^ was visually observed in the FTIR spectrum, which corresponded to the stretching vibration caused by a large amount of primary amine (-NH_2_) in PD. Therefore, dopamine successfully underwent oxidative self-polymerization on the CF surface. The presence of different degrees of the induction effect and the conjugation effect of dopamine during oxidation resulted in the FTIR peaks of all functional groups in Figure 14b shifting to different degrees.

**Figure 15:**
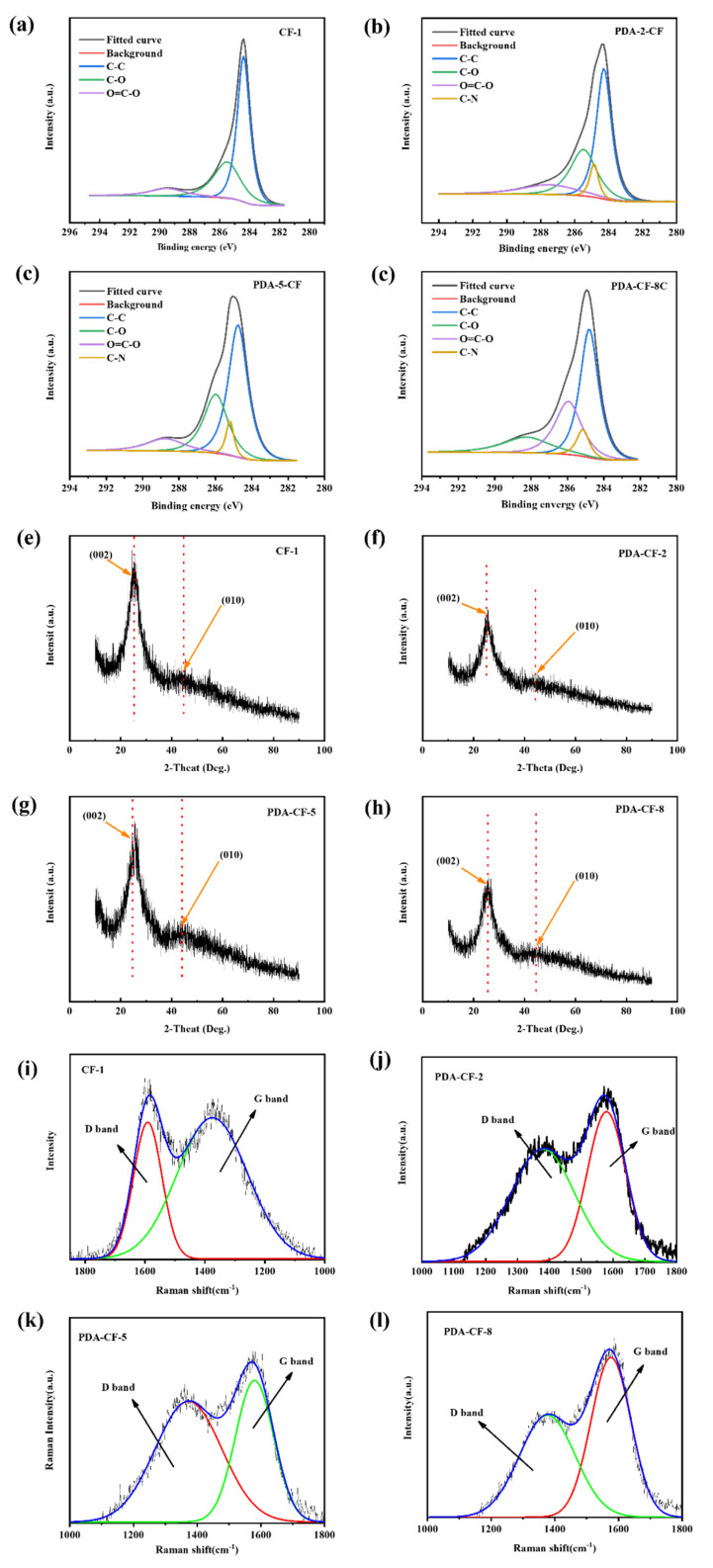
C1s XPS peak fit of CF-1 (**a**), PDA-2-CF (**b**), PDA-5-CF (**c**), and PDA-8-CF (**d**). Raman spectrum of CF-1 (**e**), PDA-2-CF (**f**), PDA-5-CF (**g**), and PDA-8-CF (**h**). (**i**) XRD pattern of CF-1 (**i**), PDA-2-CF (**j**), PDA-5-CF (**k**), and PDA-8-CF (**l**).

It can be clearly observed from Figure 15a-d that the carbon fibers treated with dopamine showed new C-N peaks after C1s fitting, and the C-O peak as well as the O=C-O peak area of the carbon fibers were significantly changed. This was attributed to the fact that the dopamine molecule contained functional groups such as hydroxyl (OH) and amine (-NH_2_) groups, which could chemically react with the functional groups on the surface of carbon fibers to form C-O bonds and C-N bonds. For example, hydroxyl functional groups could undergo esterification or condensation reactions with carboxylic acid or carbonyl functional groups on the surface of carbon fiber to form C-O bonds; amine functional groups could undergo condensation reactions with acidic functional groups on the surface of carbon fiber to form C-N bonds; and some surface active centers might be formed during the adsorption of dopamine on the surface of carbon fiber, which had certain catalytic effects and promoted the formation of C-O bonds, C=O bond, and C-N bond formation. Furthermore, the dopamine molecule itself had an oxidative nature and could oxidize with the functional groups on the surface of carbon fibers to form C=O bonds. This oxidation reaction might involve the reaction of the aldehyde group (CHO) or ketone group (C=O) in the dopamine molecule with the functional groups on the surface of the carbon fiber.

As shown in Figure 15e–h, the CF had a high carbon content. The first-order Raman spectrum of the original CFs exhibited two typical peaks at 1330–1340 cm^−1^ (D-band) and 1590–1600 cm^−1^ (G-band), which corresponded to the degree of carbon amorphization and graphitization, respectively. Meanwhile, the integrated areas of the D-band and G-band were calculated separately [46], and the degree of graphitization of CFs was subsequently measured using the area ratio (ID/IG). Raman spectra were subjected to split-peak fitting processing using the Lorentzian function to obtain parameters such as the position, half-peak width, and peak intensity of the D-band and G-band characteristic peaks. By calculating the peak fitted integral area ratio of the samples, it was found that the ID/IG of CF-1 was 1.03, while the ID/IG after dopamine modification was 0.72 (PDA-CF-2), 0.74 (PDA-CF-5) and 0.71 (PDA-CF-8), respectively. Additionally, the area ratio presented a significant decreasing trend, and the area ratio significantly decreased, probably because, after dopamine modification, the aliphatic structure of the CF was formed because of the reaction between the carboxyl group on the CF and the amino group on PD. Furthermore, after dopamine modification, the organic groups on the surface of the CF were reduced. Dopamine could change the molecular orbitals of carbon atoms in CFs from sp^3^ orbitals to sp^2^, thereby improving the graphitization of CFs to some extent. In addition, the aromatic ring structure in dopamine interacted with the aromatic structure on the carbon fiber surface by π-π stacking, which promoted the formation of sp^2^ hybridized carbon structure on the carbon fiber surface. This stacking action could also increase the aromatic carbon content on the carbon fiber surface, and the functional groups in dopamine (such as amino and phenolic groups) underwent chemisorption reactions with the functional groups on the carbon fiber surface to form chemical bonds. This chemisorption could further introduce new sp^2^ hybridized carbon structures, and the stacking effect as well as various effects such as chemical reactions led to a significant increase in the sp^2^ degree of the carbon fibers [23,26]. Therefore, the intensity of the G-peak of the modified CF increased in the spectra, which also yielded a significant increase in the peak of the -CH- stretching vibration at 2860 cm^−1^ in the FTIR spectra (Figure 14b). Additionally, the D-band peak exhibited material defects. As the content of PD on the CF surface increased, the degree of surface defects decreased, and the intensity of the D-peak decreased correspondingly.

As shown in Figure 15i, the characteristic diffraction peaks of the untreated CFs were observed at 2θ = 25.9° and 43.1° in the XRD pattern, and the diffraction peaks corresponding to (002) and (010) of PDA-CF (Figure 15j–l) with different sizing concentrations were in the same position as the two diffraction peaks of CF-1, demonstrating PD as an amorphous polymer. The sized dopamine could undergo oxidative self-polymerization on the CF surface and protect the CF crystal structure without changing the properties of the CFs.

## 4. Conclusions

Herein, by modifying CFs with different lengths and preparing corresponding CF papers, the effect of dopamine-modified CFs on CF paper was studied. A green CF modification method was obtained from the experimental data analysis, and the CF modified using this method prepared carbon fiber paper with uniform fiber dispersion, low resistivity, and high permeability. The tensile strength of the CF paper prepared using modified PDA-CF was four times higher (3 mm and 6 mm), and the modified CFs improved the paper uniformity of the CF paper. The close contact between the fibers reduced the electron transfer resistance. The resistivity of the CF paper after dopamine modification reached as low as 0.13536 Ω·cm^−1^ (3 mm) and 0.06457 Ω·cm^−1^ (6 mm), which were about 32.6% and 32.93% lower than the CFP-0, respectively. Additionally, dopamine oxidation was observed on the CF surface. PD attached to the CF had little effect on the CF permeability, and compared with the virgin CF paper, the maximum reduction in permeability was only 8.17% (3 mm and 6 mm). Furthermore, the use of dopamine-modified CFs in the preparation of carbon fiber paper greatly expanded the application of modified carbon fibers, and the successfully prepared PDA-CFP could be used as a good substrate layer for GDL and further loaded with other conductive substances to produce excellent GDL.

## Figures and Tables

**Table 1:** Ra and Rq of the CFs.

Sample	Ra	Rq
CF-0	29.8 nm	37.5 nm
CF-1	58.8 nm	70.1 nm
PDA-2-CF	39.5 nm	53.4 nm
PDA-5-CF	41.2 nm	54.7 nm
PDA-8-CF	53.9 nm	68.3 nm

## Data Availability

Not applicable.
